# Association of serum YKL-40 and DPP4 with T2-high asthma in Chinese adults

**DOI:** 10.1097/MD.0000000000037169

**Published:** 2024-02-09

**Authors:** Li Zhang, Liang Li, Mei Zhou, Qian-Yun Zhou, Ji-Hong Tang, Mei Liang, Qin Liu, Xiao-Feng Fu

**Affiliations:** a Department of Respiratory and Critical Care Medicine, The People’s Hospital of Yubei District of Chongqing City, Chongqing, China; b Department of Clinical Laboratory, The People’s Hospital of Yubei District of Chongqing City, Chongqing, China.

**Keywords:** association, DPP4, FeNO, T2-high asthma, T2-low asthma, YKL-40

## Abstract

This study aimed to assess the utility of serum YKL-40 and serum dipeptidyl peptidase IV (DPP4) as biomarkers for distinguishing between type 2 (T2)-high and T2-low asthma in the Chinese population. Additionally, we sought to explore the associations of serum YKL-40 and DPP4 levels with asthma characteristics and conventional markers. A real-world observational cross-sectional study was conducted, involving a total of 75 adult asthma patients. We collected general information, including demographics and medical history. Measurements included complete blood count, fractional exhaled nitric oxide (FeNO), post-bronchodilator spirometry, serum YKL-40 and serum DPP4 levels. Asthma endotypes, T2-high and T2-low, were defined through a comprehensive review of existing literature and expert group discussions. Logistic and linear regression models were employed. Our findings indicated no significant association between serum YKL-40 or serum DPP4 levels and T2-high asthma across all models. In the fully adjusted model, their odds ratios (OR) were 0.967 (95% CI: 0.920–1.017) and 0.997 (95% CI: 0.993–1.001), respectively. Notably, serum YKL-40 exhibited a positive correlation with FeNO (β = 0.382, 95% CI: 0.230–0.533) after adjusting for confounding factors. This association, however, diminished in patients under 40 years old (*P* = .24), males (*P* = .25), and those with FEV1%pred of 80% or higher (*P* = .25). Serum DPP4 demonstrated a negative correlation with FEV1/FVC in the fully adjusted model (β: −0.005, 95% CI: −0.009, −0.000). Among Chinese adult asthma patients, a positive correlation was observed between serum YKL-40 levels and FeNO in females aged over 40 with FEV1%pred less than 80%. Additionally, a weak negative correlation was found between serum DPP4 levels and FEV1/FVC. However, neither serum YKL-40 nor serum DPP4 levels exhibited the capability to differentiate between T2-high and T2-low asthma.

## 1. Introduction

Asthma is a heterogeneous disease with varying underlying pathogenic mechanisms.^[1]^ Consequently, precise categorization of asthma holds promise for facilitating improved personalized treatment, particularly in the context of biologically targeted medications and steroid therapy.^[2–5]^ Based on indicators such as fractional exhaled nitric oxide (FeNO), blood eosinophils, other type 2 inflammatory biomarkers, and atopy, patients with asthma can be divided into 2 distinct endotypes: T2-high asthma and T2-low asthma.^[1,6]^ In recent years, new biomarkers, notably YKL-40 and dipeptidyl peptidase IV (DPP4), have emerged, demonstrating close associations with asthma.^[7–9]^ Further exploration of these promising biomarkers may lead to advancements in asthma classification and personalized treatment strategies.

YKL-40, a human chitinase-like protein encoded by the CHI3L1 gene, plays a significant role in the inflammatory response of asthma, especially in airway remodeling and tissue fibrosis.^[10–13]^ Previous studies demonstrated that patients with asthma exhibit a significant elevation in serum YKL-40.^[14]^ However, the relationship between circulating YKL-40 and asthma endotype remains inconsistent. Initial studies indicated an association between YKL-40 and type 2 airway inflammation,^[15–17]^ whereas research suggested a link between YKL-40 and non-type 2 airway inflammation.^[18,19]^ It was noteworthy that YKL-40 is influenced by CHI3L1 single nucleotide polymorphism,^[13,20]^ indicating the potential for variation in YKL-40 expression levels among different racial groups. Yet, there was a lack of research exploring the relationship between YKL-40 and asthma endotype in the Chinese population. Therefore, one of the aims of this study was to explore whether serum YKL-40 levels were associated with T2-high asthma, asthma features or conventional markers in the Chinese population.

DPP4, a glycoprotein produced by interleukin-13 (IL-13) induced bronchial epithelial cells, is highly expressed in the bronchial epithelial cells of untreated asthma patients.^[21]^ DPP4 is implicated in airway inflammation and detectable in serum, rendering it a potential biomarker for guiding targeted anti-IL-13 therapies.^[22]^ The association between serum DPP4 levels and response to anti-IL-13 targeted therapy has been demonstrated in a phase IIb study of tralokinumab.^[23]^ IL-13 functions as a pivotal cytokine in type 2 airway inflammation, implying that DPP4 should theoretically exhibit an association with type 2 airway inflammation as well. Nevertheless, existing literature has failed to establish a link between serum DPP4 and type 2 inflammatory biomarkers, including FeNO, blood and sputum eosinophils, and Immunoglobulin E.^[22]^ Until now, it was unclear if serum DPP4 can effectively distinguish asthma endotype. Another goal of this study was to examine the association between serum DPP4 levels and T2-high asthma, along with asthma features or conventional markers in the Chinese population.

## 2. Methods

### 2.1. Study design

A real-world, observational, cross-sectional study was conducted. The data were collected from stable asthma patients, referring to those who were not experiencing acute exacerbation,^[24]^ at the outpatient department of The People’s Hospital of Yubei District of Chongqing City. This study strictly adhered to the principles outlined in the Helsinki Declaration and was approved by the Ethics Committee of the People’s Hospital of Yubei District of Chongqing City (Approval Number. (2021) (SA04)). All participants signed written informed consent forms. This study was registered with the Chinese Clinical Trial Registry on March 15, 2022, under registration number ChiCTR2200057627.

### 2.2. Participants

Data from 75 adult patients with asthma were collected continuously between March 2022 and October 2022. The diagnosis of asthma was based on the GINA guidelines.^[1]^ Inclusion criteria consisted of individuals aged 18 years or older, absence of acute asthma exacerbation at enrollment, and regular use of asthma control medication for a minimum of 3 months. Conversely, the exclusion criteria included the presence of acute infectious diseases, such as pneumonia; concurrent major chronic conditions like chronic obstructive pulmonary disease, cardiovascular or cerebrovascular ailments, diabetes, and mental disorders; notable anomalies in liver, kidney, or thyroid function; pregnancy or lactation status; and patient or familial refusal to participate in the study.

### 2.3. General information collection

General data of all participants were collected in the form of a questionnaire, including sex, age, height, weight, detailed history of asthma and medication usage, asthma attacks in the past year, atopy status including history of specific allergen sensitivities and allergic rhinitis, family history of asthma, smoking status, asthma symptom control levels, and asthma control test (ACT) score. The questionnaire survey was conducted independently by 2 respiratory physicians to ensure data reliability.

### 2.4. Measurement of blood cell count

Peripheral blood samples were collected using anticoagulant tubes and immediately subjected to a 5-category routine blood test using a Beckman Coulter DxH-800 Analyzer. The absolute values of eosinophils and neutrophils were expressed as cells/μL.

### 2.5. Measurement of serum levels of YKL-40 and DPP4

Peripheral blood samples were collected in anticoagulant-free tubes and allowed to stand for 30 minutes. Subsequently, the samples were centrifuged at 1500 rpm for 15 minutes at 4°C. The obtained serum samples were stored at −70°C for further analysis. Commercial ELISA kits (Elabscience Biotechnology Co. Ltd., Wuhan, Hubei Province, China) were used to quantify the serum levels of YKL-40 and DPP4. The minimum detection limits for YKL-40 and DPP4 were 37.5 pg/mL and 0.47 ng/mL, respectively. The samples were subjected to different dilutions for testing, and the concentrations of YKL-40 and DPP-4 were derived through curve fitting; both were expressed in ng/mL.

### 2.6. Measurements of FeNO and lung function

FeNO was measured using the RuiBreath N1®, a portable hand-held NO analyzer (RuiBreath AB, Guangzhou, China), following international guidelines.^[25]^ Post-bronchodilator spirometry, including forced expiratory volume in the first second/forced vital capacity (FEV1/FVC) and forced expiratory volume in the first second % predicted (FEV1%pred), was performed using the MasterScreenPFT device (Jaeger, Bavaria, Germany), in accordance with the recommendations of the European Respiratory Society.^[26]^

### 2.7. Definition of T2-high asthma and T2-low asthma

The most reliable method for distinguishing between T2-high and T2-low asthma required obtaining airway epithelium through invasive procedures. However, in clinical practice, performing bronchoscopic biopsies to identify T2 signaling pathways from the airway epithelium in every patient was impractical.^[27]^ Hence, in this clinical study, based on a review of previous literature and expert group discussions, we defined T2-high and T2-low asthma using FeNO, eosinophil count, and clinical characteristics.^[1,28,29]^ Patients were categorized as having T2-high asthma if their blood eosinophil count was ≥ 300/μL or FeNO was ≥ 50ppb. Additionally, T2-high asthma also included individuals with blood eosinophil count ranging from 150/μL to < 300/μL, or FeNO levels between 25ppb and < 50ppb, along with a confirmed atopy status, which included a history of specific allergen sensitivities or allergic rhinitis. Those who did not meet these criteria were categorized as T2-low asthma.

### 2.8. Statistical analyses

Continuous variables were presented as mean ± SD or median (Q1–Q3), while categorical variables were expressed as percentages. For continuous variables, we compared the characteristics of subjects using the Kruskal–Wallis H test. For categorical variables, we compared the characteristics of the subjects using the chi-square test or Fisher’s precision probability test. Subsequently, logistic regression models were employed to assess the association between serum levels of YKL-40 and DPP4 with T2-high asthma. The OR and 95% confidence intervals (CI) were used to estimate this possibility. Three models were developed: an unadjusted model with no covariate adjustments; a minimally adjusted model adjusting for age and sex; and a fully adjusted model accounting for age, sex, body mass index (BMI), smoking status, blood eosinophils, blood neutrophils, and FeNO. Univariate analysis was conducted to explore the variables potentially associated with YKL-40 and DPP4 levels. Following the identification of correlations between YKL-40 and FeNO as well as between DPP4 and FEV1/FVC, linear regression models were employed to further elucidate their relationships. The beta coefficient (β) and 95% CI were used to represent the relationships between them. Additionally, smooth curve fitting and threshold effect analysis were established to assess the presence of any non-linear relationship. Stratified analysis was used to assess the consistency of the relationship between the serum YKL-40 and FeNO levels. Forest plot was applied to better present the results of stratified analysis. All statistical analyses were performed using R software (http://www.R-project.org) and EmpowerStats (http://www.empowerstats.com), with the significance level set at *P* < .05.

## 3. Results

The demographic and clinical characteristics of the 75 participants were summarized in Table 1. Asthmatic patients were categorized into 2 groups: the T2-high asthma group (n = 46) and the T2-low asthma group (n = 29), as predefined. Compared to the T2-low asthma group, the T2-high asthma group exhibited higher FeNO levels (*P* < .001), a greater blood eosinophil count (*P* < .001), and a higher percentage of atopic status (history of specific allergen sensitivities, *P* < .001; allergic rhinitis, *P* = .007). These measures contributed to the definition of T2-high asthma in this study. Furthermore, the T2-high asthma group had lower ACT scores (*P* < .001) and FEV1%pred values (*P* = .007). Within the T2-high asthma group, the median serum levels of YKL-40 and DPP4 were 38.8 ng/mL (Q1–Q3: 25.7–66.9) and 1076.4 ng/mL (Q1–Q3: 968.6–1227.0), respectively. Meanwhile, the T2-low asthma group showed median serum levels of YKL-40 and DPP4 at 30.9 ng/mL (Q1–Q3: 17.0–44.4) and 1105.5 ng/mL (931.4–1444.8), correspondingly. In summary, there were no statistically significant differences in serum levels of these 2 biomarkers between the 2 groups. Similarly, no statistically significant differences were observed among the 2 groups in terms of age, sex, BMI, education, smoking status, asthma history in close relatives, asthma symptom control levels, number of asthma attacks in the past year, FEV1/FVC and blood neutrophil count (*P* > .05).

**Table 1:** Characteristics of patients according to asthma endotype grouping.

Variable	T2-low asthma (n = 29)	T2-high asthma (n = 46)	*P* value
Age (y, mean ± SD)	49.5 ± 13.4	46.3 ± 14.9	.349
Sex			
Male, n (%)	11 (37.9)	18 (39.1)	.917
Female, n (%)	18 (62.1)	28 (60.9)	
BMI (kg/m^2,^ mean ± SD)	23.1 ± 2.7	22.1 ± 2.9	.176
Education			
Elementary school graduation, n (%)	7 (24.1)	5 (10.9)	.423
Junior high school graduation, n (%)	7 (24.1)	10 (21.7)	
High school graduation or equivalent, n (%)	7 (24.1)	16 (34.8)	
College or above, n (%)	8 (27.7)	15 (32.6)	
Smoking status			
No, n (%)	22 (75.9)	39 (84.8)	.334
Yes, n (%)	7 (24.1)	7 (15.2)	
Specific allergen sensitivities			
No, n (%)	28(96.5)	21 (45.6)	<.001
Yes, n (%)	1 (3.5)	25 (54.4)	
Asthma history in close relatives			
No, n (%)	19 (65.5)	27 (58.7)	.555
Yes, n (%)	10(34.5)	19 (41.3)	
Allergic rhinitis			
No, n (%)	26 (89.7.7)	28 (60.9)	.007
Yes, n (%)	3 (10.3)	18 (39.1)	
Asthma symptom control			
Well control, n (%)	8 (27.6)	6 (13.0)	.200
Partly control, n (%)	15 (51.7)	24 (52.2)	
Uncontrolled, n (%)	6 (20.7)	16 (34.8)	
ACT score (mean ± SD)	21.2 ± 2.5	18.5 ± 3.9	.001
Number of asthma attacks in the past year (median (Q1–Q3))	1.0 (0–2.0)	1.0 (1.0–2.0)	.052
FEV1/FVC (%, mean ± SD)	69.5 ± 11.3	66.7 ± 10.6	.217
FEV1%pred (%, mean ± SD)	85.6 ± 18.4	75.9 ± 18.2	.007
FeNO (ppb, median (Q1–Q3))	30.0 (23.0–38.0)	58.0 (48.0–80.5)	< .001
Blood eosinophils (cells/μL, median (Q1–Q3))	130.0 (100.0–160.0)	322.5 (210.0–480.0)	<.001
Blood neutrophils (cells/μL, median (Q1–Q3))	4170.0 (3760.0–5220.0)	3730.0 (3325.0–4427.5)	.064
DPP4 (ng/mL, median (Q1–Q3))	1105.5 (931.4–1444.8)	1076.4 (968.6–1227.0)	.261
YKL-40 (ng/mL, median (Q1–Q3))	30.9 (17.0–44.4)	38.8 (25.7–66.9)	.053

ACT = asthma control test, BMI = body mass index, d = day, DPP4 = serum dipeptidyl peptidase IV levels, FeNO = fractional exhaled nitric oxide, FEV1%pred = forced expiratory volume in the first second %predicted, FEV1/FVC = forced expiratory volume in the first second/forced vital capacity, n = number, Q1 = first quartile, Q3 = third quartile, SD = standard deviation, y = year, YKL-40 = serum YKL-40 levels.

Logistic regression models were employed to further explore the relationship between the serum levels of YKL-40 and DPP4 with T2-high asthma. The models were shown in Table 2. In accordance with the guidelines outlined in the Strengthening the Reporting of Observational Studies in Epidemiology (STROBE) statement,^[30]^ the findings from unadjusted, minimally adjusted and fully adjusted analyses were simultaneously displayed. All models indicated no significant association between serum YKL-40 and T2-high asthma, as well as serum DPP4 and T2-high asthma. In the fully adjusted model, their OR were 0.967 (95% CI: 0.920–1.017) and 0.997 (95% CI: 0.993–1.001), respectively. These results suggested that serum YKL-40 and DPP4 levels cannot distinguish between T2-high and T2-low asthma.

**Table 2:** Relationship between serum levels of YKL-40 and DPP4 with T2-high asthma in different models.

Variable	Model 1		Model 2		Model 3	
	OR (95% CI)	*P* value	OR (95% CI)	*P* value	OR (95% CI)	*P* value
YKL-40	1.018 (0.999, 1.038)	.063	1.017 (0.998, 1.037)	.079	0.967 (0.920, 1.017)	.188
DPP4	0.999 (0.997, 1.000)	.095	0.999 (0.997, 1.000)	.106	0.997 (0.993, 1.001)	.111

Model 1 adjusted for none. Model 2 adjusted for age and sex. Model 3 adjusted for age, sex, BMI, smoking status, blood eosinophils, blood neutrophils and FeNO.

**Figure 1.**
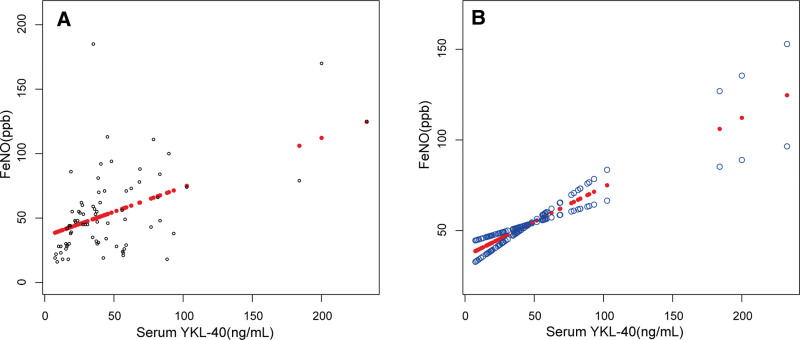
The association between serum YKL-40 and FeNO. (A) Each black point represents a sample. (B) Red dotted lines represent the smooth curve fit between variables. The area between 2 blue dotted lines is expressed as a 95% CI. Age, sex, BMI, smoking status, blood eosinophils and blood neutrophils were adjusted.

**Figure 2.**
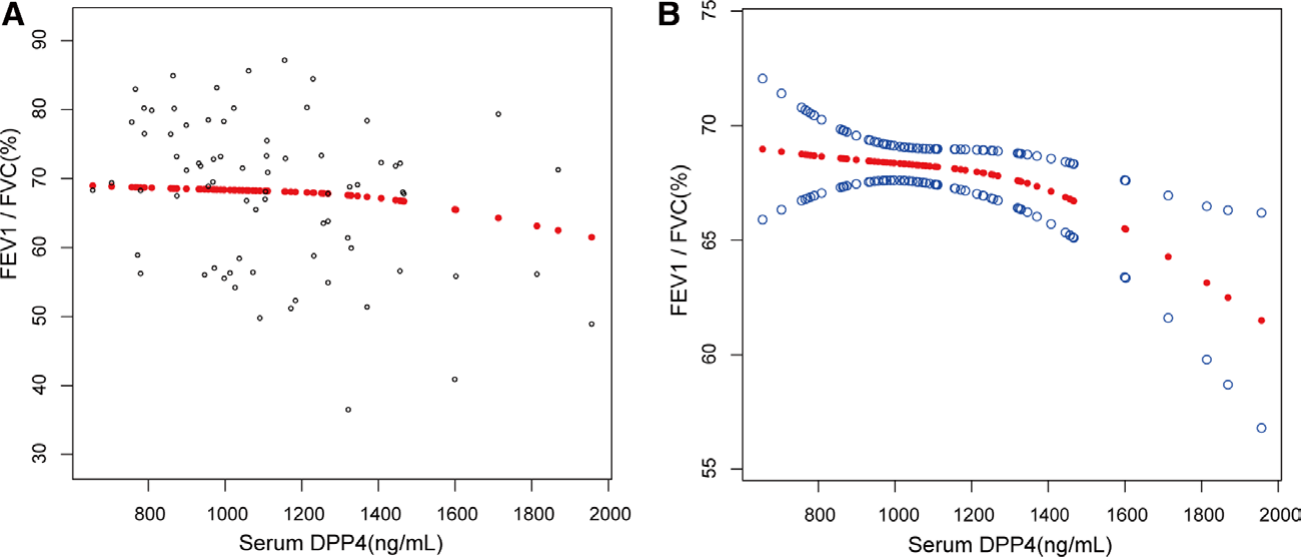
The association between serum DPP4 and FEV1/FVC. (A) Each black point represents a sample. (B) Red dotted lines represent the smooth curve fit between variables. The area between 2 blue dotted lines is expressed as a 95% CI. Age, sex, BMI, smoking status, FEV1%pred, blood eosinophils, blood neutrophils and FeNO were adjusted.

Then, univariate analyses which was adjusted for age and sex were performed with serum YKL-40 and serum DPP4 levels as dependent variables to identify the factors associated with them. The results were summarized in Table 3. FeNO levels were positively correlated with serum YKL-40 levels (*P* < .001), and FEV1/FVC was negatively correlated with serum DPP4 levels (*P* = .015). Linear regression was used to analyze the relationship between serum YKL-40 and FeNO levels, as well as between serum DPP4 and FEV1/FVC. As shown in Table 4, all 3 models consistently indicated a positive correlation between serum YKL-40 and FeNO levels, with *P* values below .001. In the fully adjusted model, β was 0.382 (95% CI: 0.230–0.533). In Figure 1, the smooth curve fit better presented the linear relationship between serum YKL-40 and FeNO. Table 5 portrayed the dynamics surrounding the negative correlation between serum DPP4 and FEV1/FVC. The negative correlation was relatively weak in the fully adjusted model (β: −0.005, 95% CI: −0.009, −0.000). In Figure 2, the smooth curve fit indicated a non-linear relationship between serum DPP4 and FEV1/FVC, prompting further threshold effect analysis. The threshold effect analysis revealed a turning point. When serum DPP4 was less than 1463.84 ng/mL, there was no significant correlation between serum DPP4 and FEV1/FVC (*P* = .871). However, above this turning point, a negative correlation was observed (*P* = .004). Details of the threshold effect are provided in Table 6.

**Table 3:** Univariate analysis for serum YKL-40 levels and serum DPP4 levels.

Covariate	Statistics	YKL-40 β (95% CI), *P* value	DPP4 β (95% CI), *P* value
Smoking status			
No	61 (81.3%)	Reference	Reference
Yes	14 (18.7%)	3.306 (−26.610, 33.222), .829	−183.926 (−381.604, 13.753), .072
BMI (kg/m^2^)	22.5 ± 2.8	−0.164 (−3.624, 3.296), .926	12.384 (−10.825, 35.593), .299
Number of asthma attacks in the past year	1.6 ± 2.0	0.161 (−4.653, 4.975), .948	−7.227 (−39.717, 25.263), .664
ACT score	19.6 ± 3.6	−1.676 (−4.224, 0.873), .202	1.283 (−16.141, 18.707), .886
FEV1/FVC (%)	67.8 ± 10.9	−0.560 (−1.552,0.433), .273	−8.259 (−14.745, 1.772), .015
FEV1%pred	80.0 ± 18.9	−0.364 (−0.875, 0.148), .168	−2.636 (−6.086, 0.813), .139
Blood eosinophils (cells/μL)	343.4 ± 445.9	0.016 (−0.005, 0.037), .132	0.002 (−0.142, 0.147), .977
Blood neutrophils (cells/μL)	4149.9 ± 1211.6	0.001 (−0.007, 0.008), .882	0.035 (−0.016, 0.086), .182
FeNO (ppb)	53.5 ± 32.5	0.654 (0.409, 0.898), <.001	−0.978 (−2.911, 0.955), .325

Adjusted for age and sex.

**Table 4:** Relationship between serum YKL-40 levels and FeNO in different models.

Variable	Model 1		Model 2		Model 3	
	β (95% CI)	*P* value	β (95% CI)	*P* value	β (95% CI)	*P* value
YKL-40	0.429 (0.272, 0.585)	<.001	0.426 (0.267, 0.586)	<.001	0.382 (0.230, 0.533)	<.001

Model 1 adjusted for none. Model 2 adjusted for age and sex. Model 3 adjusted for age, sex, BMI, smoking status, blood eosinophils, blood neutrophils.

**Table 5:** Relationship between serum DPP4 levels and FEV1/FVC in different models.

Variable	Model 1		Model 2		Model 3	
	β (95% CI)	*P* value	β (95% CI)	*P* value	β (95% CI)	*P* value
DPP4	−0.013 (−0.021, −0.004)	.004	−0.010 (−0.017, −0.002)	.015	−0.005 (−0.009, −0.000)	.035

Model 1: adjusted for none. Model 2: adjusted for age and sex. Model 3: adjusted for age, sex, BMI, smoking status, FEV1%pred, blood eosinophils, blood neutrophils and FeNO.

**Table 6:** Threshold effect analysis for the relationship between serum DPP4 and FEV1/FVC.

Models	FEV1/FVC	
	β (95% CI)	*P* value
Models I		
One line slope	−0.005 (−0.009, −0.001)	.0306
Models II		
Turning point	1463.84	
<1463.84	−0.000 (−0.006, 0.005)	.871
>1463.84	−0.019 (−0.031, −0.006)	.004
LRT test		.014

Adjusted for age, sex, BMI, smoking status, FEV1%pred, blood eosinophils, blood neutrophils and FeNO.

**Figure 3.**
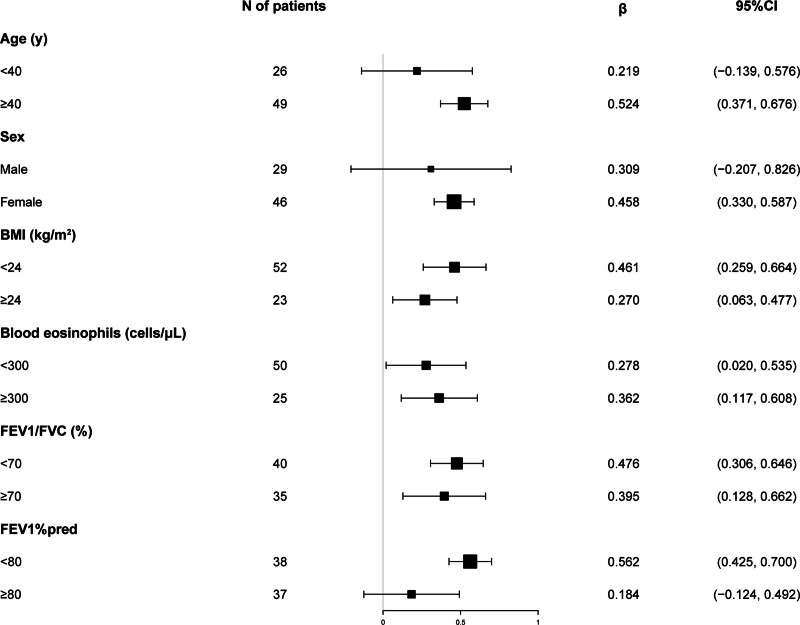
Forest plot of linear regression for the correlation between YKL-40 and FeNO, stratified by age, sex, BMI, blood eosinophils, FEV1/FVC, and FEV1%pred. Beta coefficient (β) represents the change in FeNO in ppb for a 1 ng/mL increase in YKL-40.

The aforementioned findings substantiated the correlation between serum YKL-40 and FeNO levels. Therefore, we conducted a stratified analysis within distinct subgroups delineated by age, sex, BMI, blood eosinophil count, FEV1/FVC, and FEV1%pred to delve deeper into the consistency of this relationship. Stratified assessments based on BMI, blood eosinophil count, and FEV1/FVC had no significant impact on the outcomes, as elucidated in Table 7. However, the link between serum YKL-40 and FeNO dissipated among patients under the age of 40, males, and those with an FEV1%pred of 80% or higher. In Figure 3, the forest plot illustrated the outcomes of the above stratified analysis. This observation underscored that the connection between serum YKL-40 and FeNO levels was discernible solely within specific population subsets.

**Table 7:** Effect size of serum YKL−40 levels on FeNO in prespecified subgroups stratified by age, sex, BMI, EOS, FEV1/FVC and FEV1%pred.

Characteristic	No of participants	β (95% CI)	*P* value
Age (yr)			
<40	26	0.219 (−0.139, 0.576)	.243
≥40	49	0.524 (0.371, 0.676)	<.001
Sex			
Male	29	0.309 (−0.207, 0.826)	.250
Female	46	0.458 (0.330, 0.587)	<.001
BMI (kg/m^2^)			
< 24	52	0.461 (0.259, 0.664)	<.001
≥ 24	23	0.270 (0.063, 0.477)	.018
Blood eosinophils (cells/μL)			
< 300	50	0.278 (0.020, 0.535)	.039
≥ 300	25	0.362 (0.117, 0.608)	.008
FEV1/FVC (%)			
< 70	40	0.476 (0.306, 0.646)	<.001
≥ 70	35	0.395 (0.128, 0.662)	.006
FEV1%pred			
<80	38	0.562 (0.425, 0.700)	<.001
≥80	37	0.184 (−0.124, 0.492)	.249

## 4. Discussion

The primary objective of this study was to investigate whether serum YKL-40 or serum DPP4 levels could effectively differentiate between T2-high and T2-low asthma among Chinese adults. Additionally, we aimed to identify asthma characteristics and conventional markers associated with serumYKL-40 or serum DPP4 levels. Our analysis using logistic regression models on Chinese stable asthma patients revealed that neither serum YKL-40 nor serum DPP4 levels significantly distinguished between T2-high and T2-low asthma. Furthermore, we confirmed a positive correlation between the serum YKL-40 and FeNO levels. However, upon stratified analysis, this correlation was absent among individuals under 40 years of age, males, and those with FEV1%pred > 80%. Univariate analysis showed a negative correlation between serum DPP4 and FEV1/FVC, but this correlation weakened when multiple adjusted variables were included in the linear regression model. However, this study did not find any significant correlations between serum YKL-40 or DPP4 and variables such as BMI, smoking status, or neutrophil counts.

Lee et al^[16]^ discovered that the T helper 2 (Th2) inflammatory response was weakened in mouse models with YKL-40-related gene knock out. Additionally, Specjalski et al^[31]^ revealed a positive correlation between serum YKL-40 levels and the blood eosinophil count. Another study by Tang et al^[15]^ indicated a similar positive correlation between serum YKL-40, eosinophil count, and serum Immunoglobulin E. While these preliminary investigations proposed YKL-40 as a potential biomarker for T2-high asthma, recent studies have reported contradictory outcomes. For instance, Liu et al^[32]^ unveiled that serum YKL-40 was positively correlated with blood neutrophils and negatively correlated with blood eosinophils, suggesting its potential as a biomarker for non-eosinophilic asthma. Furthermore, a distinct study indicated that YKL-40 displayed no significant associations with neutrophils and eosinophils, yet it did exhibit correlations with clinical features such as asthma exacerbation, control levels, atopy, and obesity.^[33]^ An airway transcriptome analysis in the asthma group with higher serum YKL-40 levels showed activation of non-Type 2 inflammatory pathways.^[19]^ This study also found an association between elevated serum YKL-40 levels and 2 distinct asthma phenotypes: one characterized by irreversible airway obstruction and the other by severe exacerbation.^[19]^ Building upon the cumulative evidence, Specjalski et al^[18]^ also supported the notion that YKL-40 might predominantly represent non-T2 asthma. However, our investigation revealed that elevated serum YKL-40 levels were not effective in distinguishing between T2-high and T2-low asthma. The complete role of YKL-40 in the pathogenesis of asthma remains incompletely elucidated. Therefore, the potential of YKL-40 to provide stable utility in discerning asthma endotypes or phenotypes necessitates further validation through large-scale clinical investigations and foundational research to elucidate its mechanisms.

In addition, within the subset of female asthma patients aged > 40 years with FEV1%pred < 80%, we observed a positive correlation between serum YKL-40 levels and FeNO. A previous study in pediatric patients with asthma indicated that those with elevated serum YKL-40 levels also exhibited elevated FeNO levels.^[34]^ However, another study suggested a lack of direct relationship between serum YKL-40 and FeNO.^[35]^ The inconsistencies in these outcomes might have stemmed from multifactorial influences on FeNO and the variability in serum YKL-40 levels. YKL-40 has been confirmed to be associated with airway inflammation and airway remodeling.^[35]^ A study from Japan suggested that YKL-40 is associated with the mechanism of airway remodeling in patients with severe asthma.^[11]^ Another study conducted in treatment-resistant pediatric asthma patients also indicated that YKL-40 is associated with bronchial wall thickening.^[34]^ On the other hand, a study has linked FeNO to bronchial wall thickening in asthma patients, indicating a potential role for FeNO measurement in assessing airway structural changes.^[36]^ Therefore, it is reasonable to speculate that the correlation between serum YKL-40 and FeNO may be attributed to their involvement in potential mechanisms of airway structural alterations. However, further research is needed.

Despite the recent emphasis on investigating the relationship between YKL-40 and asthma, there remains a current lack of well-defined reference values for serum YKL-40, which could be used for asthma diagnosis or to distinguish its phenotypes and endotypes. Additionally, elevated serum YKL-40 levels have been observed in various conditions, including chronic obstructive pulmonary disease, tumors, and autoimmune diseases,^[18]^ raising skepticism about its significance in asthma. Therefore, reevaluating the true value of YKL-40 in the context of asthma deserves careful consideration.

IL-13 was found in low concentrations in the serum, and the serum IL-13 levels in asthma patients resemble those of healthy volunteers.^[37]^ As a result, serum IL-13 currently lacks practical applicability in clinical settings. Meanwhile, DPP4 is induced by IL-13 in bronchial epithelial cells and can be easily measured in the bloodstream.^[21,22,38]^ Brightling et al^[23]^ showed that serum DPP4 could be a potential biomarker for targeted therapy of IL-13. As a crucial cytokine in asthma, IL-13 participates in the Th2 inflammatory response. Hence, DPP4 was postulated as a potential biomarker for T2-high asthma. However, in this study, we observed that serum DPP4 levels did not play a role in distinguishing between T2-high and T2-low asthma. Additionally, serum DPP4 showed no correlation with T2-inflammatory biomarkers like FeNO and blood eosinophils. Similar findings were overviewed by Emson et al,^[22]^ indicating a lack of association between serum DPP4 and several T2 inflammatory biomarkers.

A previous clinical study demonstrated that serum DPP4 levels were elevated in asthma patients, regardless of whether they were using inhaled corticosteroids, compared to healthy control groups.^[9]^ However, another study revealed that serum DPP4 levels in asthma patients were lower than those in healthy control groups, especially among patients with severe asthma taking inhaled corticosteroids.^[39]^ Hence, current clinical research findings regarding the expression of serum DPP4 in asthma patients are inconclusive. DDP4 is highly expressed in bronchial epithelial cells of asthma patients.^[21]^ However, whether circulating DPP4 can represent local concentrations lacks definitive literature support. Therefore, large-scale clinical research to explore this correlation may is needed in the future.

To date, no studies have reported the correlation between DPP4 and lung function have been found. This study also discovered a weak correlation between elevated serum DPP4 levels and lower FEV1/FVC, but threshold effect analysis suggested that this relationship was significant only at higher levels of serum DPP4. The DPP4 inhibitor sitagliptin can ameliorate airway remodeling in mice with chronic asthma by reducing the production of IL-13, inflammatory cells, and fibrotic factors such as TGF-β.^[40]^ Therefore, it is speculated that our finding of the relationship between serum DPP4 and FEV1/FVC may be associated with the impact of DPP4 on airway remodeling, subsequently influencing lung ventilatory function. However, there were only 8 patients with serum DPP4 levels equal to or above the turning point (1463.84 ng/mL). Therefore, the clinical significance of the weak correlation between serum DPP4 and FEV1/FVC obtained in the regression model was very limited. A larger-scale study involving more asthma population would be necessary to draw a reliable conclusion. Moreover, previous study has reported elevated DPP4 levels in obese patients.^[41]^ However, in this study, no mutual influence between BMI and serum DPP4 levels was observed among patients with asthma.

The significance of this study was highlighted by its pioneering discovery of a correlation between serum YKL-40 and FeNO levels in a specific subgroup of Chinese adult asthma patients. Moreover, it provided inaugural confirmation that neither serum YKL-40 nor serum DPP4 could effectively distinguish between T2-high and T2-low asthma in adult Chinese asthma patients. However, this study had certain limitations. First, the sample size was relatively small. Second, it lacked control groups comprising patients with acute exacerbations of asthma and healthy individuals. Last, there was a lack of dynamic follow-up for participants, including various indicators primarily focused on serum YKL-40 and DPP4 levels.

Owing to the heterogeneity of asthma, researchers are striving to identify highly specific, sensitive, reliable, and easily measurable biomarkers to facilitate phenotyping and guide treatment for asthma. However, in clinical practice, no single biomarker has been identified that meets these criteria. The lack of a single biomarker may stem from the heterogeneity of asthma and the complexity of its pathogenesis. Therefore, the search for asthma biomarkers may require large-scale clinical trials, the potential combination of multiple biomarkers, and further in-depth mechanistic research.

## 5. Conclusion

Among Chinese adult asthma patients, a positive correlation was observed between serum YKL-40 levels and FeNO in females aged over 40 with FEV1%pred less than 80%. Additionally, a weak negative correlation was found between serum DPP4 levels and FEV1/FVC. However, neither serum YKL-40 nor serum DPP4 levels exhibited the capability to differentiate between T2-high and T2-low asthma.

## Acknowledgments

Thanks to Fei Chen for her role in statistical analysis and manuscript editing.

## Author contributions

**Conceptualization:** Li Zhang.

**Data curation:** Li Zhang, Liang Li, Qian-Yun Zhou.

**Formal analysis:** Li Zhang, Liang Li.

**Funding acquisition:** Li Zhang.

**Investigation:** Li Zhang, Mei Zhou, Qian-Yun Zhou, Ji-Hong Tang, Mei Liang.

**Methodology:** Li Zhang, Liang Li, Qin Liu.

**Project administration:** Li Zhang, Xiao-Feng Fu.

**Resources:** Li Zhang, Liang Li, Qin Liu.

**Supervision:** Mei Zhou, Xiao-Feng Fu.

**Writing – original draft:** Li Zhang.

**Writing – review & editing:** Li Zhang, Liang Li.
